# A systems approach reveals that the musculoskeletal tissues development and homeostasis network

**DOI:** 10.1186/ar3583

**Published:** 2012-02-09

**Authors:** Hiroshi Asahara

**Affiliations:** 1 Department of Systems BioMedicine, Tokyo Medical and Dental University, Bunkyo-ku, Tokyo, 113-8519, Japan; 2 Department of Systems BioMedicine, National Research Institute for Child Health and Development, Setagaya-ku, Tokyo, 157-8535, Japan; 3 The Scripps Research Institute, La Jolla, CA, 92037, USA; 4 JST, CREST, Chiyoda-ku, Tokyo, 102-0075, Japan

## Materials and methods

We created a whole-mount in situ hybridization database, termed EMBRYS http://embrys.jp/embrys/html/MainMenu.html, containing expression data of 1520 transcription factors and cofactors expressed in E9.5, E10.5, and E11.5 mouse embryos --a highly dynamic stage of skeletal myogenesis. This approach implicated 43 genes in regulation of embryonic myogenesis, including a transcriptional repressor, the zinc-finger protein RP58 (also known as Zfp238) [1].

## Results

Knockout and knockdown approaches confirmed an essential role for RP58 in skeletal myogenesis. Cell-based high-throughput transfection screening revealed that RP58 is a direct MyoD target. Microarray analysis identified two inhibitors of skeletal myogenesis, Id2 and Id3, as targets for RP58-mediated repression. Consistently, MyoD-dependent activation of the myogenic program is impaired in RP58 null fibroblasts and downregulation of Id2 and Id3 rescues MyoD's ability to promote myogenesis in these cells.

## Conclusions

Our combined, multi-system approach reveals a MyoD-activated regulatory loop relying on RP58-mediated repression of muscle regulatory factor inhibitors. We applied our systems approaches to other locomotive tissues research including cartilage and tendon, and revealed novel molecular network regulating joint cartilage development and homeostasis via microRNA-140 [2,3] and tendon development by Mkx [4].
